# Therapeutic potential of *Origanum vulgare* leaf hydroethanolic extract against renal oxidative stress and nephrotoxicity induced by paraquat in rats

**DOI:** 10.22038/AJP.2019.13466

**Published:** 2019

**Authors:** Ali Sharifi-Rigi, Esfandiar Heidarian

**Affiliations:** 1 *Clinical Biochemistry Research Center, Basic Health Sciences Institute, Shahrekord University of Medical Sciences, Shahrekord, Iran *

**Keywords:** Antioxidant, Kidney, Nephrotoxicity, Paraquat, Oxidative stress, TNF-α

## Abstract

**Objective:**

Paraquat is a herbicide with potent toxicity in humans and animals. This study aimed to evaluate the protective effects of *Origanum vulgare* (*O. vulgare*) leaf extract on the acute nephrotoxicity and renal oxidative stress caused by paraquat.

**Materials and Methods:**

We randomly assigned forty male rats into five groups (G1-G5). The G1 was used as control; G2 only received paraquat (25 mg/kg body weight (bw)/day, *po*); and G3, G4 and G5 received 25 mg/kg b.w/day oral doses of paraquat and *O. vulgare* hydroethanolic leaf extract (200, 400, 800 mg/kg bw/day, *po*, respectively). After 2 weeks, superoxide dismutase (SOD), renal catalase (CAT), vitamin C levels, histopathological changes, and tumor necrosis factor-α (*TNF-α*) gene expression as well as serum levels of urea, creatinine (Cr), and protein carbonyl (PC) were determined.

**Results:**

In G2, oral administration of paraquat significantly increased (p<0.05) serum Cr, urea, PC, and renal *TNF-α* gene expression relative to those of the control group. Renal catalase, superoxide dismutase, and vitamin C levels were decreased significantly (p<0.05) in G2 as compared to G1. Administration of *O. vulgare* leaf extract not only increased the renal vitamin C, CAT, and SOD but also decreased the renal *TNF-α* gene expression, malondialdehyde (MDA), serum urea and creatinine in paraquat-induced nephrotoxicity in rats.

**Conclusion:**

Our results show that *O. vulgare* leaf extract has protective effects against nephrotoxicity induced by paraquat in rats. It seems that the nephroprotective effects of *O. vulgare *extract may be related to its antioxidant and anti-inflammatory effects.

## Introduction

Paraquat is a commonly used non-selective herbicide (Li et al., 2015). This herbicide’s chemical composition is 1, 1-dimethyl-4, 4-bipyridinium dichloride. It is a very toxic herbicide to both humans and animals. There are many reports of death due to accidental exposure to paraquat in humans, which can occur due to unavailability of an effective treatment (Atashpour et al., 2017; Han et al., 2014; Hu et al., 2017). Paraquat can damage some human organs including the lung, kidney, and heart (Liu et al., 2017). This herbicide spreads out rapidly in different body tissues. Paraquat can accumulate within the kidneys at high concentrations. Also, paraquat-induced nephrotoxicity is one of the leading causes of the paraquat-induced death (Wei et al., 2014). Renal tubules lose their regular shapes when exposed to paraquat and paraquat can induce congestion of kidney blood vessels and degeneration of glomeruli (Gu et al., 2016). Paraquat exerts its herbicide action by preventing reduction of NADP^+^ to NADPH during photosynthesis (Atashpour et al., 2017). In mammals, this herbicide is converted to paraquat radical by NADPH-oxidases. Then, it passes its extra electrons to molecular oxygen and forms reactive oxygen species (ROS) such as superoxide anion (O_2_^-^), hydroxyl radical (HO^-^), and hydrogen peroxide (H_2_O_2_). ROS induce oxidative stress and damage to DNA, proteins, lipids, and disruption of the cell structure and function (Awadalla, 2012; Charão et al., 2015; Han et al., 2014; Malekinejad et al., 2010). Also, superoxide anion causes lipid peroxidation and cell death by attacking membrane’s unsaturated lipids (Atashpour et al., 2017).

Antioxidants are compounds which can prevent oxidative stress (Mittler, 2002). *Origanum vulgare* (*O.* *vulgare*) is a globally well-known aromatic herb widely used in the western diets as a spice (Savković et al., 2016; Zhang et al., 2014). *O.* *vulgare* is a Mediterranean herb from Lamiaceae family with anti-carcinogenic, anti-mutagenic, and antimicrobial properties (Kubatka et al., 2016). The important phenolic compounds of *O. vulgare *with antioxidant properties are ursolic acid, rosmarinic acid, caffeic acid, and carnosic acid (Kaurinovic et al., 2011; Pahlavan et al., 2013). The protective effects of *O. vulgare *on gentamicin-induced nephrotoxicity were confirmed in a previous study in a rat model (Mirzaei et al., 2016). Therefore, based on the above-mentioned properties of* O. vulgare*, this study sought to investigate the effects of *O. vulgare* leaf extract on renal superoxide dismutase (SOD), catalase (CAT), vitamin C, malondialdehyde (MDA) levels, tumor necrosis factor-α (TNF-α) gene expression as well as serum levels of creatinine (Cr), protein carbonyl (PC), and urea in paraquat-induced renal toxicity in rats.

## Materials and Methods

**Chemicals**

Paraquat (paraquat dicholoride, 20% purity) was obtained from Shandong Luba Chemical Co. Ltd., Jinan, China. Blood urea and creatinine (Cr) kits were prepared from Pars Azmoon Company (Tehran, Iran). SYBR^®^ Green polymerase chain reaction (PCR) Master Mix was purchased from Qiagen (Düsseldorf, Germany). Sodium acetate and thiobarbituric acid were provided by Merck Co. (Darmstadt, Germany). Nitro blue tetrazolium, riboflavin, 2, 4, 6-tripyidyl-s-triazine, and vitamin C were obtained from Sigma-Aldrich company (St. Louis, Mo USA). All other chemicals were of analytical grade. 

**Herbs and extraction procedure**

Medical Plants Research Center of Isfahan University of Medical Sciences, Isfahan, Iran kindly provided us with necessary amount of *O. vulgare*. Also, a voucher specimen was deposited (herbarium No. 502). *O. vulgare* leaves were air-dried at ambient temperature and ground to fine powder. Then, *O. vulgare*’s hydroalcoholic extract was prepared through mixing the powder in a solution of ethanol and water (70:30, v/v), at ambient temperature for 2 days. The resulting solution was carefully filtered and dried using a rotary evaporator at 50C. The resulting extract was stored at 5°C for future use.

**Measuring antioxidant, flavonoid, and phenolic contents**

The methods described by Chang et al. and McDonald et al. were used for determining the antioxidant capacity and the total phenolic content of *O. vulgare* leaf extract (Chang et al., 2002; McDonald et al., 2001).

**Animal treatment and experimental design**

We used forty 10-12 week old male Wistar rats weighting about 180-220 g. Rats were kept under standard laboratory conditions (22±2°C, 60±5% humidity, and 12:12 light/dark cycle) during the study period with free access to standard rat pellet diet and water. These animals were divided randomly into five groups of eight each. Group 1 (control group) only received oral distilled water for 2 weeks. Group 2 were treated by oral paraquat (25 mg/kg body weight (bw)/day) through gastric gavage for 2 weeks (Akinloye et al., 2013). Groups 3, 4, and 5 received oral paraquat (25 mg/kg bw/day) and treated with oral *O. vulgare* hydroethanolic leaf extract (200, 400, and 800 mg/kg bw/day, respectively) at an interval of 1 h for 2 weeks.

Thereafter, rats were anesthetized using chloroform, cardiac puncture procedure was used for collecting blood specimens, and serum and plasma were separated. Also, we collected kidney sample for determination of *TNF-α* gene expression, CAT and SOD levels, and histopathological examinations. All procedures were conducted following approval of the Ethics Committee of Shahrekord University of Medical Sciences, Shahrekord, Iran (Ethic number IR. SKUMS. REC. 1395. 151).

**Biochemical analysis**

Enzymatic assessment using auto analyzer system (BT 3000, Rome, Italy) was done for measurement of urea and Cr. Serum TNF-α was measured by enzyme-linked immune-sorbent assay (ELISA) kit (Bioassay technology laboratory Shanghai, China).

**Determination of serum and renal malondialdehyde (MDA) levels**

Serum and renal MDA levels were determined as discussed previously (Heidarian and Soofiniya, 2011).

**Measurement of ferric reducing ability of plasma (FRAP)**

Plasma antioxidant capacity of the experimental groups were assessed using Heidarian and Soofiniya (2011) protocol.

**Determination of renal catalase (CAT) and superoxide dismutase (SOD) activities**

Renal catalase activity was measured as described previously (Heidarian et al., 2014). The renal SOD activity was explored using renal tissue samples by Beauchamp and Fridovich (1971) method. Bradford method was used for measuring total protein content (Bradford, 1976).

**Determination of renal vitamin C levels**

We measured renal vitamin C level in the experimental groups through application of Omaye et al. method (Omaye et al., 1979). A standard curve for vitamin C was prepared using a concentration range of 0-20 μg/μl.

**Determination of** ***TNF-α*** **gene expression**

Real-Time quantitative PCR (RT- qPCR) and the ΔΔCT method were used for studying the expression of *TNF-α* gene (Valipour et al., 2016). *β-actin* was used as the internal control and its mRNA expression level was used for normalization of data. The following primers were prepared for determination of *TNF-α* and *β-actin* expression: *TNF-α* forward: 5´-CTGGCGTGTTCATCCGTTC-3´, reverse: 5´ GGCTCTGAGGAGTAGACGATAA-3´ and *β-actin* forward: 5'-CTTCTACAATGAGCTGCGTGTGGCC-3', reverse: 5'-GGAGCAATGATCTTGATCTTCATGG-3'.

**Determination of serum protein carbonyl (PC)**

The Reznick and Parker's spectrophotometric method was used for measurement of serum PC level (Reznick and Packer, 1994). 

**Histopathological studies of renal tissue samples**

Formalin 20% was used for fixation of renal tissue samples for further histopathological examinations. Fixed samples were embedded in paraffin and then, 5 μm thick sections were prepared for histopathologic examinations after staining by hematoxylin-eosin (H&E) (Carleton et al., 1980). The stained samples were observed under an optical microscope for any histological changes.

**Statistical analysis**

Data was analyzed using descriptive (mean±SD) and inferential (one-way ANOVA) statistics by SPSS 20.0 software package (SPSS Inc., Chicago, IL, USA). Moreover, multiple comparisons were made by Tukey's *post hoc* test. Values of p<0.05 were considered significant.

## Results

**Effects of** ***O. vulgare*** **leaf extract** **on serum levels of urea, creatinine, MDA as well as plasma FRAP**

**Figure 1:**
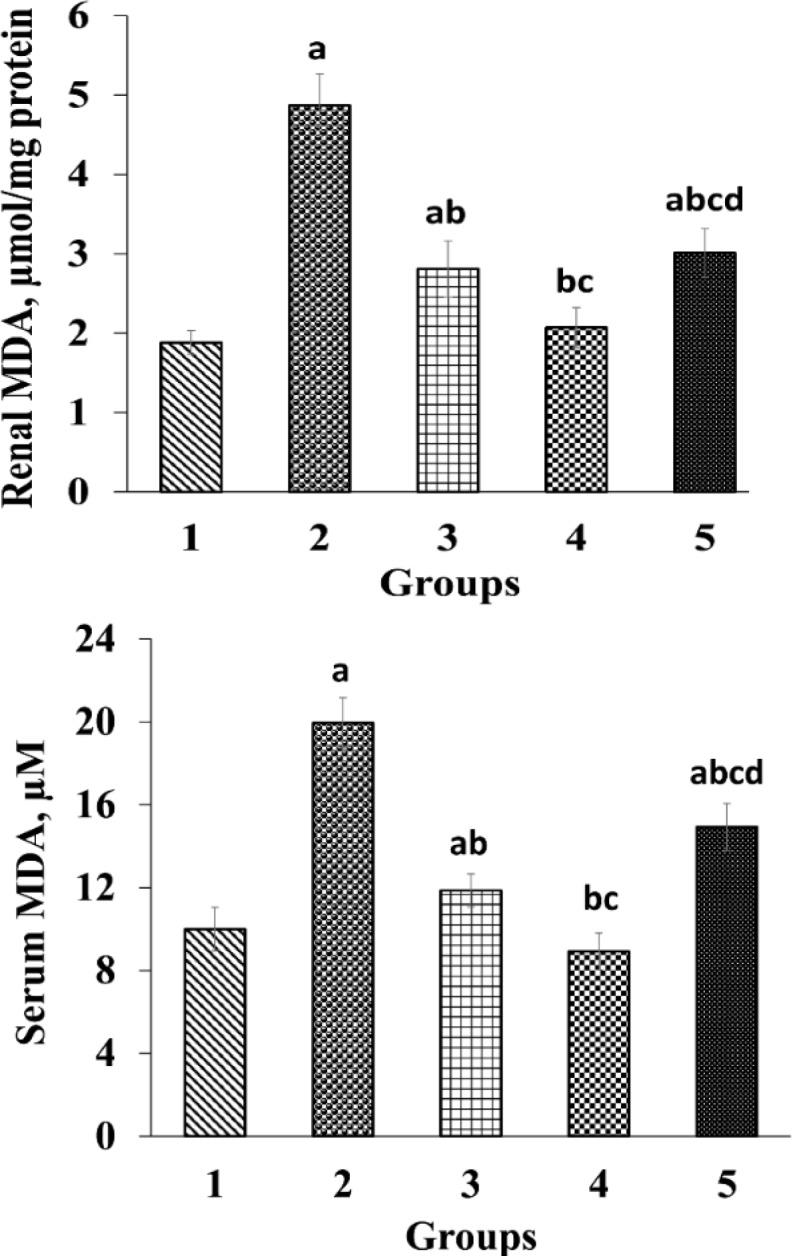
Effects of *O. vulgare* leaf extract on renal and serum malondialdehyde (MDA). Values are expressed as mean±SD. Group 1, normal control; group 2, received paraquat only; and group 3, 4 and 5, rats received paraquat and *O. vulgare* leaf extract 200, 400, and 800 mg/kg bw/day, respectively

^a^p<0.05 compared to group 1.

^b^p<0.05 compared to group 2.

^c^p<0.05 compared to group 3.

^d^p<0.05 compared to group 4.

The effects of paraquat and *O. vulgare* leaf extract on serum levels of urea, creatinine, and MDA, and renal MDA levels in the experimental groups are provided in Table 1 and Figure 1. Administration of paraquat to group 2 (treated only with paraquat) led to a remarkable increase (p<0.05) in serum levels of urea, creatinine, serum and renal MDA levels in comparison with those of G1 (the control group). Results showed a significant decrease (p<0.05) in serum levels of urea, creatinine, serum and renal MDA levels in groups treated with 200, 400, and 800 mg/kg of *O. vulgare* leaf extract relative to those of G2. Nevertheless, administration of 800 mg/kg *O. vulgare* showed a meaningful increase (p<0.05) in serum and renal MDA values relative to rats treated with 200 and 400 mg/kg (Figure 1). Also, we observed a significant reduction (p<0.05) in MDA level of G4 than G3 (Figure 1). 

**Table 1:** Effects of *O. vulgare* leaf extract on serum urea, and creatinine, and ferric reducing ability of plasma (FRAP) in paraquat-induced renal injury

**Parameters**	**Group 1**	**Group 2**	**Group 3**	**Group 4**	**Group 5**
**Creatinine (mg/dL)**	0.42±0.04	0.66±0.05^a^	0.42±0.04^b^	0.41±0.03^b^	0.44±0.04^b^
**Urea (mg/dL)**	45.37±3.81	67.50±2.07^a^	52.35±2.76^a,b^	47.12±3.22^b,c^	55.61±2.99^a,b,d^
**FRAP (µM)**	518.38±36.14	393.13±29.06^a^	537.12±42.70^b^	691.63±48.83^a,b,c^	508.75±34.70^b,d^

Values are expressed as mean±SD. Group 1, normal control; group 2, received paraquat only; and group 3, 4 and 5, rats received paraquat and *O. vulgare* leaf extract 200, 400, and 800 mg/kg bw/day, respectively.

^a^p<0.05 compared to group 1.

^b^p<0.05 compared to group 2. ^c^p<0.05 compared to group 3. ^d^p<0.05 compared to group 4.

The plasma levels of FRAP in groups treated with different doses of *O. vulgare* leaf extract (G3-G5 groups), were significantly higher (p<0.05) than G2. Moreover, the highest level of plasma FRAP was observed in the group which treated with 400 mg/kg *O. vulgare* leaf extract (Table 1). In G2 group, FRAP level had a significant reduction (p<0.05) than G1 group (Table 1). 

**Effects of** ***O. vulgare*** **leaf extract on renal CAT and SOD activities**

**Figure 2:**
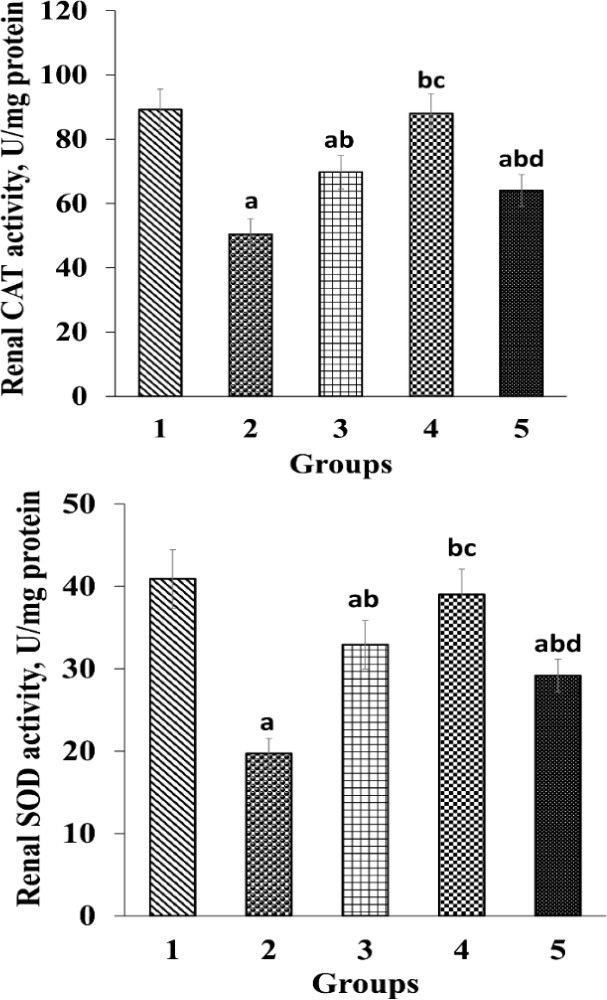
Effects of *O. vulgare* leaf extract on renal catalase (CAT), and superoxide dismutase (SOD) activities. Values are expressed as mean±SD. Group 1, normal control; group 2, received paraquat only; and group 3, 4 and 5, received paraquat and *O. vulgare* leaf extract 200, 400, and 800 mg/kg bw/day, respectively

^a^p<0.05 compared to group 1.

^b^p<0.05 compared to group 2.

^c^p<0.05 compared to group 3.

^d^p<0.05 compared to group 4.

There were significant reductions (p<0.05) in renal CAT and SOD activities in G2 compared to G1 (Figure 2). Oral administration of *O. vulgare* led to an increase (p<0.05) in renal CAT and SOD activities in G3 and G4 compared to G2. Moreover, there was a significant elevation (p<0.05) in renal CAT and SOD activities in G 5 compared to G 2. However, in G5, renal SOD and CAT activities showed a significant reduction in comparison with G 4 (Figure 2).

**Effects of** ***O. vulgare*** **leaf extract on the renal levels of vitamin C and serum levels of PC**

**Figure 3:**
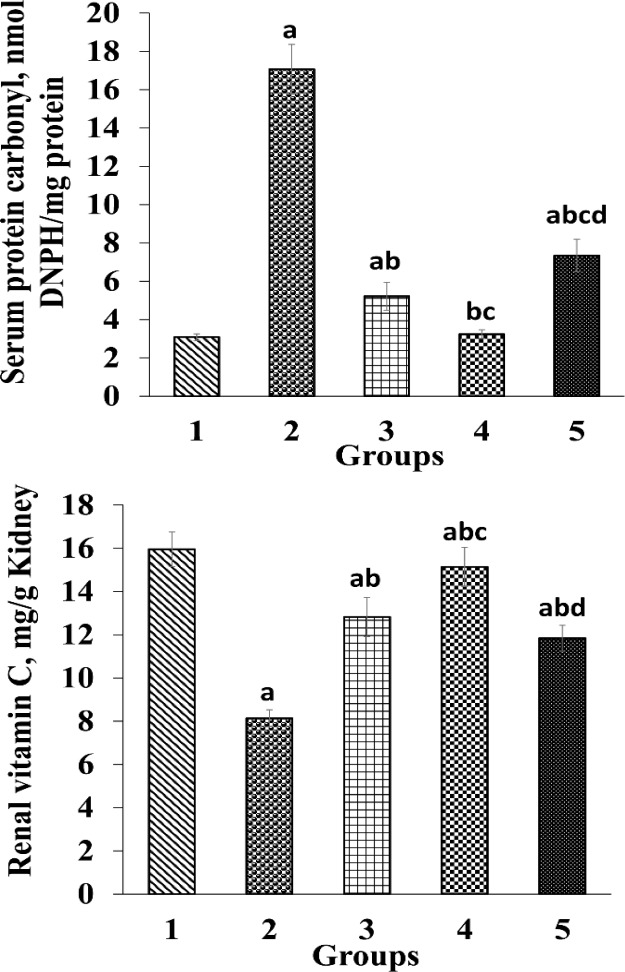
Ameliorative effects of *O. vulgare* leaf extract on serum protein carbonyl (PC) and renal vitamin C levels. Values are expressed as mean±SD. Group 1, normal control; group 2, received paraquat only; and group 3, 4 and 5, received paraquat and *O. vulgare* leaf extract 200, 400, and 800 mg/kg bw/day, respectively

^a^p<0.05 compared to group 1.

^b^p<0.05 compared to group 2.

^c^p<0.05 compared to group 3.

^d^p<0.05 compared to group 4.

The effects of *O. vulgare* leaf extract on the renal levels of vitamin C and serum PC levels are illustrated in Figure 3. Oral administration of paraquat led to a remarkable decrease (p<0.05) in renal vitamin C level in G2 compared to the control group. Furthermore, administration of different doses of *O. vulgare* leaf extract significantly (p<0.05) raised the renal levels of vitamin C compared to G2. The highest vitamin C level was observed in G 4 which was treated with 400 mg/kg *O. vulgare* leaf extract. The value of vitamin C showed a significant decrease in G 5 when compared with G4. Also, there was a significant rise in serum PC levels in G2 compared to the control group (Figure 3). Nevertheless, administration of *O. vulgare* leaf extract remarkably reduced (p<0.05) the serum PC level in comparison with G 2. Furthermore, the serum PC levels in G5 were significantly higher those that of G3 and G4 (Figure 3).

**Effects of O.** ***vulgare*** **leaf extract on serum and renal** ***TNF-α*** **gene expression**

**Figure 4:**
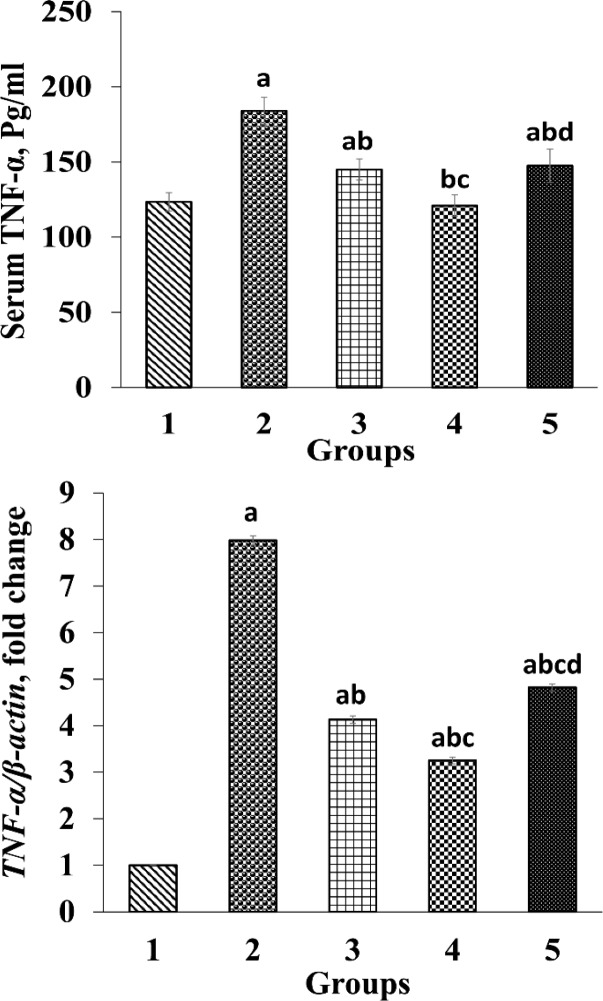
Ameliorative effects of *O. vulgare* leaf extract on serum tumor necrosis factor-α (TNF-α) and expression of TNF-α gene. Values are expressed as mean±SD. Group 1, normal control; group 2, received paraquat only; and group 3, 4 and 5, received paraquat and *O. vulgare* leaf extract 200, 400, and 800 mg/kg bw/day, respectively

^a^p<0.05 compared to group 1.

^b^p<0.05 compared to group 2.

^c^p<0.05 compared to group 3.

^d^p<0.05 compared to group 4.

The effects of *O. vulgare* leaf extract on serum and renal TNF-α levels are shown in Figure 4. There was a significant increase (p<0.05) in serum TNF-α levels and its renal gene expression in G2 in contrast to control group. Oral administration of *O. vulgare* leaf extract at different concentrations could significantly reduce (p<0.05) serum and renal *TNF-α* gene expression compared to G2. There was a meaningful decrease (p<0.05) in serum TNF-α levels and renal *TNF-α* gene expression in rats which received 400 mg/kg *O. vulgare* leaf extract as compared to those treated with 200 and 800 mg/kg doses (Figure 4).

**Histopathological findings**

**Figure 5:**
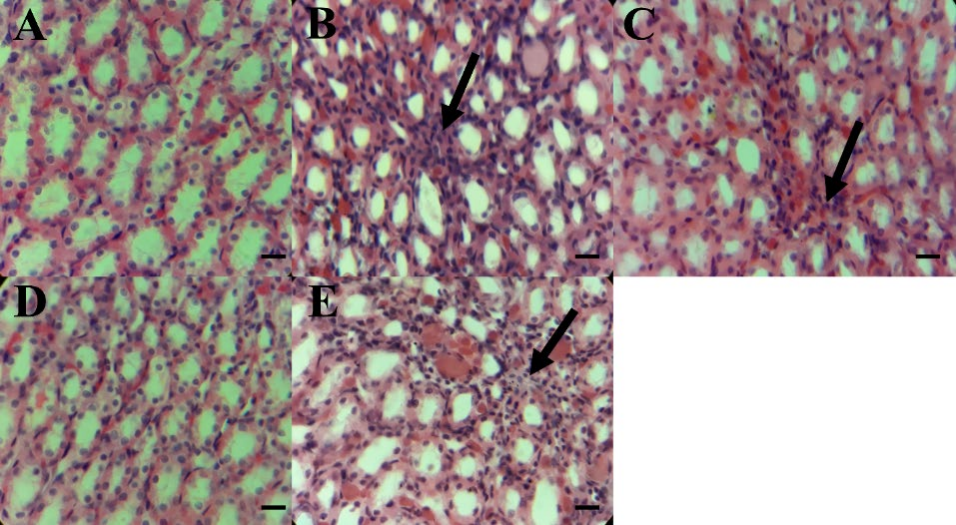
Effects of *O. vulgare *leaf hydroethanolic extract on renal histopathological changes in rats. A, the control group (group 1). B, rats treated with paraquat only (group 2) showing pathological changes in the kidney such as mononuclear cell infiltration. The black arrows show lymphocyte infiltration. C, D, and E, (groups 3-5) paraquat-administered rats supplemented with 200, 400, and 800 mg/kg bw/day of *O. vulgare *leaf hydroethanolic extract, respectively. Scale bar=50 µm; magnification ×400

Microscopic studies of the renal histological changes in G2 suggested a lymphocyte infiltration compared to the control group (Figures 5A and 5B). A significant reduction in lymphocyte infiltration was observed in the group treated with 400 mg/kg *O. vulgare* leaf extract (Figure 5D), relative to G2. Groups which received 200 and 800 mg/kg *O. vulgare* leaf extract (Figures 5C and 5E) showed a moderate decrease in lymphocyte infiltration. 

## Discussion

Application of paraquat in agriculture was done for the first time in 1962 (Han et al., 2014). This herbicide is considered a serious health issue in developing countries. The mortality rate following accidental exposure to paraquat poisoning, is about 50-90% in humans (Xu et al., 2017). This herbicide causes nephrotoxicity through oxidative stress, inflammation, apoptosis, and direct damage to renal tubules (Wei et al., 2014). Nowadays, medicinal herbs are used to treat many diseases such as renal diseases (Valipour et al., 2016). 

Creatine is converted by non-enzymatic cyclisation to creatinine (Cr) in human body and urea is produced as a residual component during the process of protein metabolism in the liver urea cycle. Cr and urea are considered the most important indicators of kidney function. Renal damage reduces the glomerular filtration rate (GFR) of kidneys and reduced GFR lead to increased serum Cr and urea levels (Bagalad et al., 2017). Elevation in serum Cr and urea, due to a decrease in GFR, was observed in paraquat and diazinon poisoning (Mohamed et al., 2015; Wei et al., 2014). In this study, we observed a meaningful increase in serum urea and Cr of the group received only paraquat compared to the control group (Table 1) which is consistent with previous studies (Gu et al., 2016; Mohamed et al., 2015; Wei et al., 2014). On the other hand, a previous study demonstrated the renal protective effects of *O. vulgare* leaf extract and reduction of serum urea and Cr in gentamicin-induced nephrotoxicity in rats (Mirzaei et al., 2016) which is in line with our findings (Table 1). Nevertheless, in our study, there was an increase in urea and Cr in group 5, which can indicate the side effects of *O. vulgare* leaf extract at a dosage of 800 mg/kg (Table 1). It was reported that herbal remedies can be harmful at higher doses through a wide range of mechanisms (Posadzki et al., 2013). Therefore, the serum urea and Cr elevation in group 5 may be due to higher dose of *O. vulgare* leaf extract.

Production of free radicals increases following exposure to insecticides through induction of oxidative stress which results in an imbalance in the antioxidant system (Hu et al., 2017). Paraquat can disrupt the equilibrium of biological antioxidant capacity and ROS production in the body through production of ROS and it also causes an oxidative stress (Han et al., 2014). Human body counteracts oxidative stress due to production of antioxidants and enzymatic and non-enzymatic antioxidants through several mechanisms which can protect the body against ROS. SOD and catalase are known enzymatic antioxidants which can convert superoxide anion into oxygen and H_2_O. Vitamin C is known as a non-enzymatic antioxidant (MatÉs et al., 1999). ROS is produced naturally during cellular biological reactions which is in equilibrium with the biological antioxidant systems (MatÉs et al., 1999; Pham-Huy et al., 2008; Wang et al., 2016). A previous study showed that paraquat poisoning reduces SOD, and CAT (Tan et al., 2015) which is in line with the findings of the present study. In the current study, paraquat-induced renal poisoning diminished renal CAT, SOD, and vitamin C levels in G2, which indicates increased oxidative stress. On the other hand, treatment with *O. vulgare* leaf extract increased SOD, CAT and vitamin C levels in the kidneys of treated groups (Figures 2 and 3). Phenolic compounds increase the activity of nuclear factor erythroid 2‐related factor 2 (Nrf2) that has a roll in synthesizing cellular antioxidant, including SOD (Deck et al., 2018; Pang et al., 2016). It was demonstrated that *O. vulgare* leaf extract has different phenolic compounds with antioxidant properties that can destroy oxidative agents (Kaurinovic et al., 2011). Also, phenolic compounds act as antioxidants (Aloulou et al., 2012; Dipti et al., 2003). Therefore, in the present study, the elevation of the renal vitamin C levels, and CAT and SOD activities can be resulted from, at least in part, *O. vulgare* leaf extract antioxidant properties.

Oxidative stress impairs the cell membrane and causes lipid peroxidation, which result in MDA production. MDA is produced from unsaturated fatty acids, and considered a lipid oxidation marker (Chen et al., 2016; Wang et al., 2016). Paraquat is considered a producer of free radicals and lipid oxidation (Pham-Huy et al., 2008). In this study, there was a significant elevation in serum and renal MDA levels in G2 compared to G1 (the control group) (Figure 1), which is in agreement with the findings of previous studies (Charão et al., 2015; Wei et al., 2014). On the other hand, in this study a significant decline in MDA level was observed in groups receiving *O. vulgare* leaf extract which is in line with findings of a previous study (Sikander et al., 2013). Also, in our study, administration of *O. vulgare* leaf extract led to an enhancement in plasma FRAP level in treated groups (Table 1). Therefore, it seems that *O. vulgare* leaf hydroethanolic extract not only can effectively prevent the elevation of MDA but also can increase FRAP because of its phenolic and flavonoids components.

ROS can damage proteins and alter their structures, and decrease protein enzymes. Paraquat leads to oxidation of proteins (Pham-Huy et al., 2008; Wang et al., 2016), and production of PC due to the oxidation of proteins (Karimi-Khouzani et al., 2017). In the current study, the serum PC levels increased significantly in G2 compared to the control group, which could indicate the oxidation of proteins by paraquat (Figure 3). Nevertheless, PC was decreased in groups treated with *O. vulgare* leaf extract. Therefore, preventing the elevation of PC in treated groups with *O. vulgare* leaf hydroethanolic extract may be related to the presence of phenolic and flavonoids compounds.

TNF-α is known as a pro-inflammatory cytokine which can regulate other inflammatory cytokines such as IL-8 and IL-6. Also, oxidative stress activates NF-κB, which increases TNF-α levels (Gu et al., 2016; Liu et al., 2017). Paraquat poisoning can damage kidneys’ glomeruli, tubules epithelium, and leukocytes permeability. Disturbed permeability for leukocytes is accompanied with the production of inflammatory mediators such as TNF-α. Our results suggested that administration of paraquat led to a remarkable increase in renal *TNF-α* gene expression and serum TNF-α in G2 (Figure 4) which is consistent with previous studies (Gu et al., 2016; Liu et al., 2017; Xu et al., 2017). It was reported that *O. vulgare* has anti-inflammatory and anti-tumor properties (Zhang et al., 2014). In this study, however, treatment with *O. vulgare* leaf extract significantly reduced serum level and gene expression of *TNF-α* (Figure 4) which is similar to the results of other studies (Kubatka et al., 2016; Mirzaei et al., 2016). Therefore, the decline in serum level of TNF-α and renal *TNF-α* gene expression, at least partly, proves the renal protective effects of *O. vulgare* leaf extract due to its antioxidant content. 

Our histopathologic findings showed an increase in mononuclear cell infiltration in G2 compared to the control group (Figures 5A and 5B). Several studies reported renal damage induced by paraquat (Liu et al., 2017; Wei et al., 2014). However, infiltration of mononuclear cells in renal tissue, improved significantly after treatment with *O. vulgare* leaf hydroethanolic extract relative to that of G2 (Figure 5). In group 5, however, oral administration of *O. vulgare *leaf extract caused an enhancement in mononuclear cell infiltration compared to groups 3 and 4 (Figures 5C and 5D). It is reported that, herbal therapy at higher doses can cause damages through a wide range of mechanisms (Posadzki et al., 2013). Also, a previous study indicated that increased production of free radicals can lead to destruction of cell membranes and tissue necrosis (Kapić et al., 2014). Therefore, elevation of mononuclear cells at 800 mg/kg of *O. vulgare *leaf hydroethanolic extract dose may be due to the side effects of *O. vulgare *leaf extract at higher doses. Nevertheless, in the present study, the prevention of histological changes and the protective effects of *O. vulgare *leaf hydroethanolic extract at 400 mg/kg dose against renal damage may be, at least in part, because of its flavonoids and phenolic compounds.

We did not assess the effects of *O. vulgare *leaf extract on renal tubular apoptosis/necrosis, pro-apoptotic markers, such as NF-*κ*B, Bax, p53, and down-regulated Bcl-2 expression in the current study. Therefore, we suggest more in-depth investigations of possible anti-apoptotic properties of *O. vulgare* leaf extract in future studies.

Our findings showed that hydroalcoholic extract of *O. vulgare* was effective against the chronic kidney damage, induced by administration of paraquat. Administration of* O. vulgare* leaf extract not only led to an increase in renal SOD, CAT and vitamin C levels but also its decreased renal MDA and *TNF-α* gene expression, as well as serum PC and MDA levels in paraquat-induced nephrotoxicity in rats. It seems that the nephroprotective effects of *O. vulgare* extract may be related to the plant’s antioxidant and anti-inflammatory effects.
